# Person-job fit and innovative behavior in new R&D institutions: the mediating effects of self-efficacy and job involvement on business decision-making

**DOI:** 10.3389/fpsyg.2025.1550324

**Published:** 2025-03-03

**Authors:** Ming Jiang, Feng Geng, Dechong Zhang, Chao Meng, Shaopeng Li, Yajie Peng

**Affiliations:** ^1^Henan Academy of Sciences, Zhengzhou, China; ^2^Henan Metallurgical Research Institute Limited Liability Company, Zhengzhou, China; ^3^Zhenglue Junce Group Co. Ltd., Beijing, China

**Keywords:** new R&D institutions, person-job fit, innovative behavior, self-efficacy, job involvement, chain mediation effect

## Abstract

In recent years, new R&D institutions have emerged in China, distinguished from traditional research entities by their unique structure and objectives. This study explores the impact of person-job fit on the innovative behavior of 334 researchers within these institutions. Through hierarchical regression analysis and bootstrap methods, we find that person-job fit significantly enhances innovative behavior. Furthermore, self-efficacy and job involvement partially mediate this relationship. These findings offer practical implications for managers seeking to foster innovation by aligning employees’ roles with their skills and motivations, thereby improving organizational effectiveness and supporting strategic business decisions.

## 1 Introduction

New R&D institutions have recently emerged in China as distinct entities focused on advancing scientific and technological innovation. These organizations operate as independent legal entities, engaging in scientific research, technological innovation, and R&D services. They feature diverse investment sources, modern management systems, market-oriented operational mechanisms, and flexible employment arrangements. Legally, they can be categorized as private non-enterprise units, public institutions, or enterprises. The operational model of these institutions is akin to global counterparts such as the Manufacturing Innovation Center in the United States, Fraunhofer-Gesellschaft in Germany, IMEC in Europe, and the Cavendish Laboratory in the United Kingdom (Dong and Wei, 2022).

Tailored to China’s unique context, these institutions reflect a government-oriented approach with innovative, de-administrative features (Zhang et al., 2017). The development of new R&D institutions can further optimize the layout of scientific research capabilities, strengthen the supply of industrial technology, promote the transfer and transformation of scientific and technological achievements, and foster the deep integration of scientific and technological innovation with economic and social development. In practice, China’s new R&D institutions can be traced back to the Shenzhen Tsinghua University Research Institute, which was jointly established by the Shenzhen Municipal Government and Tsinghua University in December 1996. The vigorous development phase of these institutions began after 2016. By the end of 2022, the number of new R&D institutions in China totaled 2,412 (Torch High Technology Industry Development Center and Ministry of Science and Technology, 2023). However, compared to the more than 40,000 social organizations in the field of science and technology and 400,000 high-tech enterprises in China, the scale of new R&D institutions remains in the early stage of development.

Compared with China’s traditional scientific research and innovation entities, new R&D institutions represent a relatively new concept and form of existence. Their connotation and extension are still being explored, and the existing research on these institutions mainly centers on the transformation of results, the construction of evaluation indexes, and operational risks. For example, Chen et al. (2018) developed a new Pasteur’s quadrant in line with the characteristics of scientific and technological achievements transfer and transformation for new R&D institutions in the new era, based on a theoretical model. Chang et al. (2023) utilized the soft system methodology, beginning with top-level strategy and through investigation and analysis, to extract a set of internationalization evaluation index systems for new R&D institutions containing six secondary and 22 tertiary indicators. Chen and Song (2023) offered targeted risk prevention suggestions for risk points in the top-level design, internal control management, and other aspects of institutional new R&D institutions based on enterprise risk management theory. The above results not only reveal many aspects of the management perspectives of new R&D institutions, but also provide useful inspiration for a more profound understanding of their operation mechanisms. Unfortunately, these studies mainly focus on the objective factors affecting the internal and external mechanisms of new R&D institutions, rather than on the researchers as the innovation body itself.

In recent years, driven by the influence of the geopolitical environment and the needs of national development, leveraging the advantages of the new lifting system, accelerating the development of high-quality productive forces, orderly layout of future industries, and securing innovations in frontier fields like artificial intelligence have become important aspects of future governmental work. The new R&D institutions are characterized by diversified investment bodies, a modernized management system, a market-oriented operation mechanism, and flexible employment mechanisms, enabling them to respond more adaptively to market changes and scientific and technological challenges. Through the development of new R&D institutions, scientific research capabilities can be further optimized, industrial technology supply can be strengthened, and the transfer and transformation of scientific and technological achievements can be promoted, fostering deep integration of scientific and technological innovation with economic and social development. These institutions are crucial for informed business decision-making in rapidly changing market environments. In this process, the role of scientific researchers has become increasingly prominent. They are not only the main drivers of scientific and technological innovation but also crucial engines for promoting institutional development. Thus, strengthening research on scientific researchers and gaining a deeper understanding of their needs, motives, and behavioral patterns is of great significance to enhancing the innovation capability of new R&D institutions.

Existing research indicates that a high level of job fit helps employees better adapt to work content, achieve work goals more effectively, and attain job satisfaction, thereby cultivating their intrinsic motivation and initiative, ultimately enhancing their self-efficacy and job involvement. These factors significantly impact innovative behavior, as high levels of job involvement are closely related to traits like initiative and creativity. However, there is a lack of systematic analysis of how person-job fit, self-efficacy, and job involvement specifically influence innovative behavior within new R&D institutions.

This study aims to address this gap by empirically examining how person-job fit influences innovative behavior among researchers in new R&D institutions and by validating the mediating roles of self-efficacy and job involvement. This research not only fills the gap in existing literature but also provides practical insights for the management of new R&D institutions, emphasizing the importance of aligning employees’ roles with their skills and motivations to foster innovation.

## 2 Literature review and hypothesis

### 2.1 Job fit and employee innovative behavior

Person-job fit and its impact on innovative behavior are crucial for strategic business decisions, as they influence organizational adaptability and competitive advantage in dynamic markets. Understanding how person-job fit can enhance innovative behavior is essential for both theoretical advancements and practical applications in the field of business management. Person-job fit can be defined as an employee’s judgment of the congruence between their own and the organization’s cultural values, as well as the congruence between their skills and the requirements of the job. This match includes congruence between the individual and the organization as a whole, as well as between the individual and the specific job, requiring the ideal employee to fit both the job and the organization as a whole (Cable and DeRue, 2002). In other words, job fit usually includes both supply-value matching and demand-ability matching (Wu et al., 2011). According to self-regulation theory, effective self-regulation requires individuals to utilize sufficient personal resources to cope with changes in external conditions (Baumeister and Heatherton, 1996). It has been shown that when the external environment is compatible with the individual’s needs, their resource consumption for self-regulation is reduced, which helps them to better cope with the challenges of daily life and maintain a good psychological state (Koole et al., 2012). Chou et al. (2022) investigated 128 full-time workers who had been working for more than 6 months from the perspective of motivation theory and analyzed them using a linear regression model to demonstrate the positive impact of job matching on individual performance. Hasan et al. (2021) emphasized the importance of providing good working conditions and maintaining work-life balance through the logic of social exchange theory and resource conservation theory, concluding that job fit enhances job satisfaction and ultimately organizational commitment. In summary, maintaining a positive mindset among workers, improving individual performance, and enhancing job satisfaction will all provide favorable support for innovative behaviors requiring high levels of concentration and strong work motivation. Thus, this paper proposes the following hypothesis:

H1: Person-job fit significantly positively affects the innovation behavior of researchers in new R&D institutions.

### 2.2 The mediating role of self-efficacy

The concept of self-efficacy was first introduced in 1977 by psychologist Albert Bandura, who defined it as an individual’s confidence and belief in their ability to successfully perform a task or behavior to produce a desired outcome (Bandura, 1977). Self-efficacy is a key component of an individual’s belief system, reflecting their confidence in their abilities and serving as a direct psychological incentive for individual behavioral decision-making and sustained effort (Seebauer and Babcicky, 2020). Research has shown that a high level of job fit helps employees better adapt to the work content, accomplish work goals more effectively, and easily attain job satisfaction, thus effectively cultivating their intrinsic motivation and initiative, and ultimately enhancing their self-efficacy (Akmal and Mehmood, 2022). Increased self-efficacy makes employees more inclined to set challenging goals and believe they are capable of overcoming difficulties, thereby increasing their motivation to work hard. For new R&D organizations, whose main outputs are scientific research results, such a mentality can both improve work performance and stimulate employees’ creativity and innovation ability (Bandura, 2012). Conversely, low job fit often makes employees feel that their work lacks challenges or is too difficult, leading to a loss of enthusiasm and motivation, which affects work efficiency and performance. In addition, low job fit may also challenge employees’ self-identity as they are unable to fully realize their abilities and potential (Rajper et al., 2020). This mismatch also affects the cooperative atmosphere of the team, as employees may feel that their contributions are underestimated or neglected, thus impacting team cohesion and collaborative effectiveness. Thus, this paper proposes the following hypothesis:

H2: Self-efficacy mediates the relationship between person-job fit and the innovation behavior of researchers in new R&D institutions.

### 2.3 The mediating role of job involvement

Work involvement refers to the degree of psychological and physiological participation in the work task, which reflects a person’s identification with the current work and state of engagement (Rabinowitz, 1977). It is an outward expression of how well an employee’s personal needs are being met. Cognitive evaluation theory suggests that when employees believe that job requirements match their knowledge, skills, and abilities, and that job compensation, work environment, and organizational climate align with their expectations—in other words, in a job-matching environment—they will feel joy, challenge, and a sense of accomplishment from the work itself. This intrinsic motivation will prompt employees to devote themselves more intensively to their work, forming a more ideal level of work involvement. Additionally, this intrinsic motivation will help individuals maintain a positive and satisfied emotional state, making them more likely to have a positive perception and evaluation of the work, thereby deepening their level of work involvement (Ćulibrk et al., 2018). For researchers in new R&D institutions, good working conditions can help them continue to invest their knowledge and skills in their research work and promote good teamwork, motivating them to take on more responsibilities in return for organizational support and inspiring them to work hard to achieve their goals. Conversely, insufficient intrinsic motivation and negative working conditions may reduce the degree of work engagement, affecting the output of research results.

Regarding the relationship between job involvement and innovative behavior, Wallace et al. concluded that increasing employees’ job involvement can directly affect their innovative behavior, especially in demanding and resource-intensive environments such as scientific research. Researchers are more likely to exhibit innovative behavior if they are given sufficient fairness and trust and are allowed to participate in decision-making on a relatively autonomous basis (Wallace et al., 2013). De Spiegelaere et al. (2014), using the European economic crisis as a backdrop, concluded from a survey of 927 respondents from five different industries that an unfavorable external environment tends to create a sense of insecurity in the workplace. This insecurity affects an individual’s motivation and dedication to work, subsequently reducing their level of involvement and ultimately decreasing employees’ innovative behaviors. The new R&D institutes are often loosely structured scientific research entities. If the work of these institutions makes researchers feel a lack of value and meaning, they are more likely to lose interest in R&D, which is not conducive to the development of their creativity. Thus, this paper proposes the following hypothesis:

H3: Job involvement mediates the relationship between person-job fit and the innovative behavior of researchers in new R&D institutions.

### 2.4 The chain mediation of self-efficacy and job involvement

When employees are in a job-matched work environment, they can fully utilize their professional skills and talents, resulting in better performance at work. This success enhances employees’ self-efficacy, which is their confidence in their ability to perform their jobs and accomplish their tasks. As self-efficacy increases, employees become more confident in their abilities and values, and their enthusiasm for work increases accordingly.

Furthermore, a high degree of self-efficacy will promote employees’ work involvement. Enhanced self-efficacy helps employees maintain perseverance and confidence in overcoming work difficulties (Caesens and Stinglhamber, 2014). When employees recognize their abilities, they become more focused on their work and are willing to invest more time and energy into completing their tasks. This high level of commitment not only helps to improve work efficiency but also enables employees to gain a greater sense of accomplishment and satisfaction in their work.

Finally, the level of employees’ job involvement significantly impacts their innovative behavior. Innovative behavior often requires employees to have high levels of initiative and creativity, traits that are closely related to high levels of job involvement. When employees are passionate and fully engaged in their work, they are more likely to generate new ideas and solutions that foster organizational innovation and growth. In addition, high self-efficacy makes employees more willing to try new approaches and ideas, further stimulating their innovative potential.

Based on the comprehensive analysis above, this paper proposes the following hypotheses:

H3: Self-efficacy and job involvement have a chain-mediated effect on the relationship between person-job fit and the innovation behavior of researchers in new R&D institutions.

**Figure 1:**
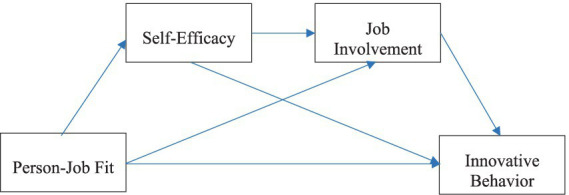
Theoretical model (*N* = 334).

Hence, the theoretical model proposed in this paper is illustrated in Figure 1.

## 3 Methodology

### 3.1 Participants and data collection

In this study, the questionnaire survey method was used to collect samples in two forms: third-party on-site or telephone interviews and anonymous online questionnaire platforms for distribution and recycling. The third-party on-site or telephone interviews collected 181 samples, accounting for 49.3% of the total, while the anonymous online questionnaire platforms collected 186 samples, accounting for 50.7% of the total. The number of new R&D organizations participating in this survey exceeds 10% of China’s current number (based on 2022 data), involving nearly 300 new R&D organizations, including the Henan Academy of Sciences, Tsinghua University Research Institute in Shenzhen, Zijinshan Laboratory, Zhijiang Laboratory, Pengcheng Laboratory, Beijing Institute of Life Sciences, and Zhangjiang Laboratory. The survey covered Henan, Shandong, Hubei, Zhejiang, Jiangsu, Guangdong, Fujian, Beijing, Shanghai, and other regions, totaling 27 provinces, municipalities, and autonomous regions. After excluding mismatched organization types, missing data, duplicate entries, and questionnaires with too short completion times, a total of 344 valid questionnaires were collected. Gender analysis revealed 238 males (69.2%) and 106 females (30.8%). In terms of age distribution: 131 researchers were within the age of 30 (38.1%), 185 researchers were between 31 and 40 years of age (53.8%), 34 researchers were between 41 and 50 years of age (7.0%), and 4 researchers were over 51 years old (1.2%). In terms of education level, 76 researchers held doctoral degrees (22.1%), 120 held master’s degrees (34.9%), 135 held bachelor’s degrees (39.2%), and 13 held other degrees (3.8%). In terms of titles, there were 176 persons with junior titles and below (51.2%), 129 persons with intermediate titles (37.5%), 30 persons with deputy senior titles (8.7%), and 9 persons with full senior titles (2.6%). In terms of years of working experience, there were 82 persons with less than 1 year of working experience (23.8%), 189 with 2–5 years (54.9%), 55 with 6–10 years (16%), and 18 with more than 11 years of experience (5.2%). The insights gained from understanding the person-job fit in these institutions can inform more effective HR strategies and business decisions, enhancing overall organizational performance.

### 3.2 Measures

To guarantee the validity of the questionnaire, the scale used in this study is an authoritative scale that has been used frequently both domestically and internationally. It adopts the Likert 5-point scoring method (Likert, 1932), with scores ranging from 5 (“fully compliant”) to 1 (“not at all compliant”). The questionnaire was adjusted according to the characteristics of new R&D institutions. Experts in human resource management from these institutions were invited to fill in the test questionnaire and provide feedback. Based on their suggestions, the questionnaire was revised and the formal survey was conducted.

1. The scale developed by Saks and Ashforth (1997) was used to measure “Person-Job Fit,” consisting of four questions, such as: “My knowledge, skills, and abilities can fulfill the requirements of the job” and “My job is exactly what I want to do.”
2. Self-efficacy was measured using a scale developed by Tierney and Farmer (2002), which contains four questions, such as: “I think I am good at drawing inspiration from other people’s ideas and developing my own set of ideas” and “I am confident in my ability to solve problems by using my creativity in the workplace.”
3. “Work Involvement” was measured using the scale of Kanungo (1982), which consists of 10 questions, such as: “Important things that happen to me are often related to my current job” and “I have strong feelings about my current job and it is difficult for me to leave it.”
4. “Innovative Behavior” was measured using a one-dimensional scale adapted following cultural validation protocols (Liu and Shi, 2009) based on Scott and Bruce’s “Innovative Behavior Scale,” which consists of five questions, such as: “In order to realize my concept or idea, I will find ways to get the resources I need to make it happen” and “I will actively develop appropriate plans or programs to implement my innovative ideas.”

### 3.3 Data analysis

In this study, SPSS 27 was used to analyze the reliability of the four sets of scales (Table 1), and Cronbach’s alpha coefficients were used to examine their reliability. A Cronbach’s alpha coefficient of 0.9 or higher is considered excellent, between 0.8 and 0.9 is considered good, between 0.7 and 0.8 is considered adequate, and 0.7 or lower indicates that the scale needs revision (Phan et al., 2012). The results of the reliability analysis for the Person-Job Fit Scale, Self-Efficacy Scale, Work Involvement Scale, and Innovative Behavior Scale are as follows:

**Table 1:** Reliability test (*N* = 334).

Scale	Cronbach’s α	Reliability Classification
Person-Job Fit	0.828	Good
Self-Efficacy	0.872	Good
Work Involvement	0.850	Good
Innovative Behavior	0.899	Good

## 4 Results

### 4.1 Validity testing

In this study, exploratory factor analysis (EFA) was employed to examine the dimensionality of the scale.

Before applying the factor model analysis, the scale data were first analyzed for factor model adaptation. In general, a KMO value greater than 0.6 (Shrestha, 2021), and a *p*-value less than 0.05 (Napitupulu et al., 2017) are considered suitable for factor analysis. After analysis, the sample KMO value was 0.925, and the significance *p*-value was less than 0.001, indicating that the data from this survey passed the adaptability test.

In this paper, we used Harman’s one-factor test to conduct principal component analysis on all measurement items. Four factors with eigenvalues greater than 1 were obtained without specifying the number of factors to be extracted. The largest factor, with the largest eigenvalue when not rotated, explained 39.717% of the variance. A variance value of less than 50% indicated that it was within the reasonably permissible range (Aguirre-Urreta and Hu, 2019) The subsequent data analysis can be carried out.

In this study, the four sets of scales were analyzed using confirmatory factor analysis with Mplus 7.4 to assess goodness-of-fit measures (Table 2). The results showed that the four-factor model fit was optimal among the alternative models, indicating that the discriminant validity of the four-variable model was satisfactory. The results are summarized as follows:

**Table 2:** Confirmatory factor analysis results (*N* = 334).

model	χ^2^ /df	RMESEA	CFI	TLI
Four-factor	3.06***	0.077	0.899	0.886
Three-factor	5.39***	0.113	0.783	0.758
Two-factor	6.96***	0.132	0.703	0.672
Single factor	7.89***	0.141	0.655	0.621

Four Factor Model = Person-Job Fit + Self-Efficacy + Job Involvement + Innovative Behavior; Three Factor Model = Person-Job Fit + (Self-Efficacy + Job Involvement) + Innovative Behavior; Two-factor model = (Person-Job Fit + Self-Efficacy) + (Job Involvement + Innovative Behavior); Single factor model = (Person-Job Fit + Self-Efficacy + Job Involvement + Innovative Behavior); Parentheses indicate merged variables; * indicates *p* < 0.05, ** indicates *p* < 0.01, and *** indicates *p* < 0.001.

### 4.2 Correlation analysis

The results of the variable correlation analysis are shown in Table 3: Person-job fit showed a significant positive correlation with self-efficacy (r = 0.558, *p* < 0.01), job involvement (r = 0.424, *p* < 0.01), and innovative behavior (r = 0.512, *p* < 0.01). Self-efficacy showed a significant positive correlation with job involvement (r = 0.462, *p* < 0.01) and innovative behavior (r = 0.707, *p* < 0.01). Work involvement showed a significant positive correlation with innovative behavior (r = 0.512, *p* < 0.01).

**Table 3:** Correlation coefficients, means and standard deviations of variables (*N* = 334).

Variable	Person-Job Fit	Self-Efficacy	Job Involvement	Innovative behavior	average value	standard deviation
Person-Job Fit	1				4.245	0.595
Self-Efficacy	0.558**	1			4.259	0.555
Job Involvement	0.424**	0.462**	1		3.086	0.604
Innovative behavior	0.512**	0.707**	0.512**	1	4.100	0.611

*Indicates *p* < 0.05; ** indicates *p* < 0.01; and *** indicates *p* < 0.001.

### 4.3 Hypothesis testing

This paper adopts hierarchical regression analysis to verify the direct effect of person-job fit on the innovative behavior of researchers in new R&D institutions, constructs the direct effect model, and carries out regression analysis after incorporating gender, age, and other control variables into the equation, and the results show that person-post fit has a significant positive effect on the innovative behavior of researchers in new R&D institutions (β = 0.491, *p* < 0.001) (see Table 4), and H1 is verified.

**Table 4:** Regression analysis of man-post fit on innovation behavior of researchers in new R&D institutions (*N* = 334).

	Model 1					Model 2				
Constant	B	β	Sig.	Tol	VIF	B	β	Sig.	Tol	VIF
Sexes	−0.128	−0.097	0.069	0.975	1.025	−0.132	−0.100	0.032	0.975	1.026
Age	0.227	0.239	<0.001	0.707	1.414	0.115	0.121	0.030	0.678	1.474
Education attainment	0.013	0.017	0.783	0.686	1.458	0.005	0.007	0.899	0.685	1.459
Title	0.000	0.000	0.994	0.654	1.530	0.011	0.019	0.736	0.653	1.532
Years of experience	−0.025	−0.032	0.619	0.662	1.511	0.026	0.033	0.560	0.654	1.530
Person-job fit						0.504	0.491	<0.001	0.951	1.051
R^2^	0.065					0.294				
Adjusted R^2^	0.051					0.282				
*F*-value	4.716		<0.001			23.438		<0.001		

Tolerance (Tol): Indicates the collinearity tolerance of independent variables in the regression model, calculated as Tol = 1 − R^2^, where R^2^ is the coefficient of determination when a predictor is regressed on other variables. Tol > 0.1 indicates acceptable collinearity. Variance Inflation Factor (VIF): The reciprocal of Tol (VIF = 1/Tol), quantifying the degree of collinearity. VIF < 10 suggests no severe multicollinearity. All Tol and VIF values in this study meet the criteria, supporting model robustness.

### 4.4 Tests of mediating effects

In this paper, the relevant hypotheses are tested using the SPSS macro program PROCESS, developed by Hayes. This program is based on the Bootstrap method (Tofighi and Kelley, 2020), which has gained widespread application in recent years, to test both the general mediating role and the multiple chain mediating role.

First, self-efficacy and job involvement were used as independent mediating variables, and the Bootstrap method was applied to test their independent mediating effects with 5,000 repetitions. The results showed that person-job fit had a significant positive effect on self-efficacy (β = 0.5205, *p* < 0.001). Self-efficacy had a significant positive effect on innovative behavior (β = 0.6734, *p* < 0.001). The value of the direct effect of person-job fit on innovative behavior was 0.1747, with a 95% CI of [0.0834, 0.2660], not including 0. The mediating effect value of self-efficacy was 0.3505, with a 95% CI of [0.2772, 0.3063], not including 0. Self-efficacy played a partial mediating role, thus verifying H2. Person-job fit had a significant positive effect on job involvement (β = 0.4306, *p* < 0.001), and job involvement had a significant positive effect on innovative behavior (β = 0.3639, *p* < 0.001). The value of the direct effect of person-job fit on innovative behavior was 0.3685, with a 95% CI of [0.2725, 0.4645], not including 0. The mediating effect value of job involvement was 0.1567, with a 95% CI of [0.0989, 0.2276], not including 0. Job involvement played a partial mediating role, thus verifying H3.

Next, the chained mediation effect of self-efficacy and job involvement was tested using the Bootstrap method with 5,000 repetitions and the PROCESS Model 6 (Hayes, 2018). This model placed both mediating variables into the analysis simultaneously. The results are presented in Table 5.

**Table 5:** Results of chained mediation effect analysis (*N* = 334).

Model path	Effect	BootSE	BootLLCI	BootULCI	Test results
Aggregate effect	0.5252	0.0477	0.4314	0.6190	Statistically significant
Direct effect	0.1224	0.0082	0.0319	0.2130	Statistically significant
Overall indirect effects	0.4028	0.0433	0.3240	0.4938	Statistically significant
Path 1. X → M → Y	0.3111	0.0380	0.2413	0.3915	Statistically significant
Path 2. X → N → Y	0.0523	0.0178	0.0221	0.0920	Statistically significant
Path 3. X → M → N → Y	0.0394	0.0118	0.0195	0.0662	Statistically significant

X stands for person-job fit; M stands for self-efficacy; N stands for job involvement, and Y stands for innovative behavior. The symbol → stands for the path direction.

The model analysis test found that the overall indirect effect was the sum of the mediating effects of path 1, path 2, and path 3, with a value of 0.4028, and its 95% CI was [0.3240, 0.4938], which did not include 0. The effect was significant. The total effect of person-job fit on innovative behavior was 0.5252, with a 95% CI of [0.4314, 0.6190], not including 0, thus further supporting H1. The mediating effect value of self-efficacy in the relationship between person-job fit and innovative behavior was 0.3111, with a 95% CI of [0.2413, 0.3915], not including 0, and the effect was significant. The mediating effect value of job involvement in the relationship between person-job fit and innovative behavior was 0.0523, with a 95% CI of [0.0221, 0.0920], not including 0, and the effect was significant. The mediating effect value of self-efficacy and job involvement in the relationship between person-job fit and innovative behavior was 0.0394, with a 95% CI of [0.0195, 0.0662], not including 0. This suggests that person-job fit can positively influence the innovation behavior of researchers in new R&D institutions through the chain mediation of self-efficacy and job involvement, thus validating H4.

**Figure 2:**
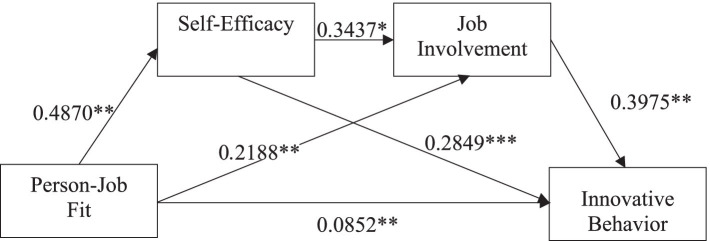
Chain mediation path of action (*N* = 334). * indicates *p* < 0.05, ** indicates *p* < 0.01, and *** indicates *p* < 0.001.

Meanwhile, this paper presents a path diagram to more intuitively illustrate the chain mediating role of self-efficacy and job involvement between person-job fit and the innovative behaviors of researchers in new R&D institutions (see Figure 2).

## 5 Discussion

New R&D institutions, products of the scientific and technological revolution and industrial change, are significant in revitalizing innovation resources, reorganizing the innovation chain, and enhancing the social innovation system’s overall effectiveness. They are expected to become vital forces in making breakthroughs in scientific and technological innovation. New R&D institutions not only bring together numerous scientific and technological elites and experts but also provide continuous innovation impetus for enterprises through advanced R&D equipment, flexible R&D mechanisms, and close cooperation between industry, academia, and research institutes.

The true potential of these new R&D institutions hinges on their ability to stimulate and unleash the innovative capacities of their researchers. The findings underscore the importance of aligning person-job fit with business strategies, as enhancing self-efficacy and job involvement can drive innovative behaviors, thereby supporting more effective business decision-making processes. This study explores the relationship between person-job fit and the innovative behavior of researchers within these institutions. Our findings indicate that person-job fit significantly enhances researchers’ innovative behavior. Moreover, self-efficacy and job involvement serve as partial mediators in this relationship, highlighting the importance of psychological and motivational factors in fostering innovation.

## 6 Theoretical implications

Filling the Research Gap: Firstly, there is limited existing research focusing on scientific researchers in new R&D institutions, particularly concerning the relationship between person-job fit and innovative behavior. This study addresses this gap by empirically verifying the relationship through model construction and hypothesis testing. By doing so, it adds systematic understanding to the study of factors influencing the innovative behavior of scientific researchers in new R&D institutions.

Clarifying Influence Pathways: Through constructing and validating the chain mediation model, this study clarifies the specific influence path of job fit on the innovation behavior of researchers in new R&D institutions. This process defines the independent and synergistic roles of self-efficacy and job involvement between job fit and innovative behavior, offering a comprehensive understanding of the influence mechanism. It provides powerful theoretical support and practical guidance for optimizing staffing and stimulating innovative vitality.

## 7 Practical implications

Importance of Person-Job Fit in Recruitment: Research has shown that mismatches between people and jobs may result in employees’ abilities and skills not fully meeting the requirements of the job. This pressure diminishes employees’ willingness and motivation to innovate, causing them to prioritize basic task completion over exploring new approaches or devising innovative solutions. Additionally, if employees feel unqualified for the job, they may lack self-confidence, further inhibiting their innovative behavior (Afsar and Masood, 2018). It is recommended that in setting job responsibilities and during the recruitment process of new R&D organizations, selection criteria should align closely with actual needs. Avoiding criteria that exceed job requirements ensures that employees are well-suited to their roles, preventing counterproductive outcomes. Ensuring a good person-job fit not only enhances employee satisfaction and innovation but also supports better business decision-making by leveraging employees’ full potential to achieve strategic goals.

Tailored Management Practices for Researchers: The work of researchers in new R&D institutions is unique as it involves independently designing experimental programs, selecting research methods, and analyzing data based on the organization’s research direction, their professional background, and experimental conditions. This personalized way of working necessitates that organizations and managers improve their management approaches based on the concept of matching people with jobs. By doing so, they can better help researchers leverage their strengths and advantages while enhancing their self-discipline and sense of responsibility.

These practical implications emphasize the need for R&D organizations to focus on optimal person-job fit and to adopt management practices that support and enhance the innovative potential of their researchers.

## 8 Limitations and prospects

Limited Sample Size: New R&D institutions are still in the construction and development stage in China. For example, in Henan Province, as of January 2024, the number of provincial-level new R&D institutions filed by the Henan Provincial Department of Science and Technology was only more than 140, resulting in a relatively small overall number of samples that can be investigated. Future studies should aim to include a larger sample size as the number of R&D institutions grows.

Data Collection Method: Approximately 50% of the data in this study were collected online through respondents’ self-assessment. Although the issue of common method bias was controlled within an acceptable range, the data source remains relatively single. Future research should attempt to use multi-source data collection methods to enhance the scientific validity and rigor of the study.

Scope of Research Content: This study focused on research personnel, but the generation of overall innovative behavior in an organization also relies on other auxiliary personnel and managers. Future research should expand the scope of the study by including personnel with different career development paths to better clarify the mechanism of the influence of person-job fit on the innovative behavior of personnel in new R&D organizations.

Addressing these limitations and expanding the scope of future research will enhance our understanding of person-job fit and its impact on innovative behavior in new R&D institutions. This will provide more comprehensive insights and practical guidance for fostering innovation in these dynamic environments.

## Data Availability

The original contributions presented in the study are included in the article/supplementary material, further inquiries can be directed to the corresponding author.
